# Comprehensive Characterization of Immune Landscape Based on Tumor Microenvironment for Oral Squamous Cell Carcinoma Prognosis

**DOI:** 10.3390/vaccines10091521

**Published:** 2022-09-14

**Authors:** Qi-Lin Li, Jing Mao, Xin-Yao Meng

**Affiliations:** 1 Department of Stomatology, Tongji Hospital, Tongji Medical College, Huazhong University of Science and Technology, Wuhan 430030, China; 2 School of Stomatology, Tongji Medical College, Huazhong University of Science and Technology, Wuhan 430030, China; 3 Hubei Province Key Laboratory of Oral and Maxillofacial Development and Regeneration, Wuhan 430030, China; 4 Department of Pediatric Surgery, Tongji Hospital, Tongji Medical College, Huazhong University of Science and Technology, Wuhan 430030, China

**Keywords:** oral squamous cell carcinoma, immunotherapy, signature, prognosis, survival

## Abstract

Objective: This study aims to identify an immune-related signature to predict clinical outcomes of oral squamous cell carcinoma (OSCC) patients. Methods: Gene transcriptome data of both tumor and normal tissues from OSCC and the corresponding clinical information were downloaded from The Cancer Genome Atlas (TCGA). Tumor Immune Estimation Resource algorithm (ESTIMATE) was used to calculate the immune/stromal-related scores. The immune/stromal scores and associated clinical characteristics of OSCC patients were evaluated. Univariate Cox proportional hazards regression analyses, least absolute shrinkage, and selection operator (LASSO) and receiver operating characteristic (ROC) curve analyses were performed to assess the prognostic prediction capacity. Gene Set Enrichment Analysis (GSEA) and Gene Ontology (GO) function annotation were used to analysis the functions of TME-related genes. Results: Eleven predictor genes were identified in the immune-related signature and overall survival (OS) in the high-risk group was significantly shorter than in the low-risk group. An ROC analysis showed the TME-related signature could predict the total OS of OSCC patients. Moreover, GSEA and GO function annotation proved that immunity and immune-related pathways were mainly enriched in the high-risk group. Conclusions: We identified an immune-related signature that was closely correlated with the prognosis and immune response of OSCC patients. This signature may have important implications for improving the clinical survival rate of OSCC patients and provide a potential strategy for cancer immunotherapy.

## 1. Background

Oral squamous cell carcinoma (OSCC) is one of the most common malignancies arising in the oral cavity worldwide, and the incidence of OSCC has been increasing by at least 1% annually, especially in China [1,2]. Common treatment options for OSCC include surgery, chemotherapy, radiotherapy, immunotherapy, and these treatment methods have reduced the mortality rate of OSCC partly [3,4]. However, despite the rapid progress in the treatment of OSCC, its mortality and incidence are still rising due to the tumor heterogeneity [5]. Therefore, in order to improve the survival rate of OSCC, the development of new predictive biomarkers to accurately predict the prognosis is of great significance for OSCC patients [6]. 

Immunotherapy is one of the recent breakthroughs in cancer therapy and becoming a new promising approach in cancer treatment, including OSCC [7,8]. However, due to tumor heterogeneity and multifaceted immunosuppressive signals within the tumor microenvironment (TME), the efficacy of cancer immunotherapy is limited [9,10]. TME refers to the cellular environment where tumor cells reside, and is composed of tumor cells, immune cells, fibroblasts, stromal cells, endothelial cells, and other components [11,12,13]. TME plays a regulatory role in tumorigenesis and in the interactions between cancer cells and surrounding components (such as cancer-associated fibroblasts, T cells, B cells, macrophages, and lymphocyte), which promotes tumor development and metastasis [8,14,15]. 

Immune and stromal cells are two most abundant cell types and their degree of infiltration in the TME has a clinical prognostic value in many cancer types [10,16,17]. Therefore, a deeper understanding of the cell components (including immune cells and stromal cells) and the immunosuppressive status of the TME is critical for improving the efficacy of immunotherapy [18]. The Estimation of Stromal and Immune cells in Malignant Tumor tissues using Expression data (i.e., ESTIMATE) algorithm is used to estimate the levels of infiltrating stromal and immune cells by calculating stromal/immune/ESTIMATE scores [19]. At the same time, the therapeutic efficacy of immunotherapy in OSCC is relatively limited. Therefore, it is crucial to identify a more individualized prognostic signature and develop a more accurate immunotherapy for patients with OSCC. Recently, immune-related signatures have been developed and used to predict prognosis in certain cancers [20], and some also have been reported in OSCC (such as those based on epithelial-mesenchymal transition, stemness of cancer, or immunosuppression genes) [2,17,21,22]. However, there is no reliable immune-related signature based on TME for predicting the prognosis of OSCC patients currently. 

In this study, the stromal and immune scores of 319 patients from the TCGA OSCC database was determined using the ESTIMATE algorithm. A total of 562 upregulated and 31 downregulated immune-related genes were identified. Moreover, a functional enrichment analysis showed that the immune-related genes mainly played a critical role in immune response, activation/proliferation of immune-related cells, and chemokine activity. Finally, a prognostic 11 genes model, i.e., *AC103563.1*, *CCL22* (C-C motif chemokine ligand 22), *FLT3* (FMS-like tyrosine 3), *GALR2* (galanin receptor 2), *IGKV1D.8*, *IGLV1.36*, *IGLV4.60*, *IL10* (interleukin 10), *LINC00861*, *LINC01508,* and *MS4A2* (membrane-spanning 4 domains, superfamily A, number 2) was constructed and confirmed to be significantly associated with the overall survival (OS) of OSCC patients. In conclusion, we screened the immune-related signature and explored the relationship between the screened immune-related signature and the prognosis of OSCC patients, and we aimed to use this signature as a potential prognostic biomarker and immune therapeutic target for OSCC patients.

## 2. Methods

### 2.1. OSCC Datasets Acquisition and Handing

The RNA-sequence dataset (TCGA, OSCC, *n* = 319) was obtained from the National Cancer Institute GDC Data Portal (https://portal.gdc.cancer.gov/) (accessed on 3 December 2020). Patients with complete follow-up data and survival status information were included in this analysis. Immune and stromal scores were calculated using the ESTIMATE algorithm [19].

### 2.2. Screening of Differentially Expressed Genes (DEGs)

The package “limma” in R (version 4.0.2) was used to screen for differentially expressed genes (DEGs) between OSCC tissue and normal tissues. FDR < 0.05 and |log2FoldChange| > 1 was considered as threshold values for the identification of DEGs. A heatmap was used to visualize the expression profiles of the DEGs. Venn diagrams were used to show the overlaps among the DEGs [23].

### 2.3. Functional Enrichment Analyses and Functional Annotation of DEGs

Gene Ontology (GO), including biological processes (BPs), molecular functions (MFs), and cellular components (CCs) were determined using the R package “ggplot2”, “clusterProfiler” and “enrichplot”, respectively. The Kyoto Encyclopedia of Genes and Genomes (KEGG) was used for the enrichment analysis of pathways [24]. The Search Tool for Retrieval of Interacting Genes/Proteins (STRING) (https://string-db.org) (accessed on 3 December 2020) was used to predict the functional interaction of proteins. Cytoscape software was used to analyze the interaction scores of the PPI network nodes, and scores > 0.95 were considered the key PPI nodes [25].

### 2.4. Construction of the Immune-Related Prognostic Signatures

OSCC patients (*n* = 319) were divided into high-risk and low-risk groups based on immune and stromal scores estimated by ESTIMATE. The Cox proportional hazards regression model was used to predict the OS of OSCC patients. *p* < 0.05 was statistically significant. Univariate Cox proportional hazards regression analyses were used to analyze the survival-related DEGs. The survival-related DEGs were put into the Cox proportional hazards model survival analysis with a least absolute shrinkage and a selection operator (LASSO) penalty. Finally, the TME-related prognostic signature was constructed by weighting the Cox regression coefficients to calculate a risk score for each patient. Using the cut-off value with the median value of the risk score, patients were classified as low-risk and high-risk.

### 2.5. Statistical Analysis

Univariate and multivariate Cox proportional hazards regression analyses were performed using the R package “survival”. A LASSO Cox survival analysis was performed using the R package “glmnet”, and a 1000-fold cross-validation was used. The R package “time-ROC” and “time AUC” were used to determine the ROC curves and area under the curve (AUC) for the predictive ability of the TME-related signatures. A heatmap was used to visualize the expression profiles of the DEGs. The alluvial diagrams were analyzed using “ggalluvial” in R. *p*-value < 0.05 was considered to be statistically significant and R software (New Jersey, USA, version 3.6.2) was used to perform the statistical analysis. The R code used in this study was showed in Text S1.

## 3. Results

### 3.1. Stromal and Immune Scores Were Correlated to Clinical Features of OSCC Patients

**Figure 1:**
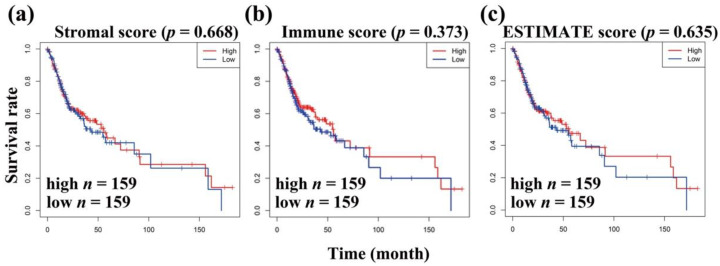
The relationship between stromal/immune/ESTIMATE scores and survival rate of OSCC patients. (**a**) KM survival analysis of OSCC patients based on their stromal scores. (**b**) KM survival analysis of OSCC patients based on their immune scores. (**c**) KM survival analysis of OSCC patients based on their ESTIMATE scores. Red line represents the survival curve of OSCC patients with higher values of stromal scores, immune scores, and ESTIMATE scores (*n* = 159). Blue line represents the survival curve of OSCC patients with lower values of stromal scores, immune scores, and ESTIMATE scores (*n* = 159).

**Figure 2:**
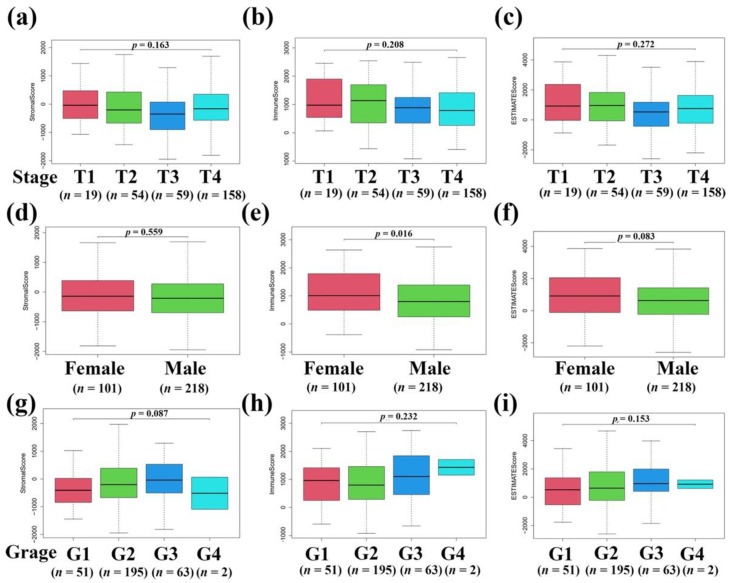
The relationship between stromal/immune/ESTIMATE scores and clinical features of OSCC patients. (**a**) Relationship between stromal scores and TNM stages of OSCC patients. (**b**) Relationship between immune scores and TNM stages of OSCC patients. (**c**) Relationship between ESTIMATE scores and TNM stages of OSCC patients. Sample numbers of each group are as follows: T1, *n* = 19; T2, *n* = 54; T3, *n* = 59; T4, *n* = 158. (**d**) Relationship between stromal scores and genders of OSCC patients. (**e**) Relationship between immune scores and genders of OSCC patients. (**f**) Relationship between ESTIMATE scores and genders of OSCC patients. Sample numbers of each group are as follows: female, *n* = 101; male, *n* = 218. (**g**) Relationship between stromal scores and tumor grades of OSCC patients. (**h**) Relationship between immune scores and tumor grades of OSCC patients. (**i**) Relationship between ESTIMATE scores and tumor grades of OSCC patients. Sample numbers of each group are as follows: G1, *n* = 51; G2, *n* = 195; G3, *n* = 63; G4, *n* = 2.

The clinical data of 319 patients with OSCC downloaded from the OSCC-TCGA RNA-seq database were analyzed in the present study. To explore the relationship between stromal and immune scores and the clinical characteristics of OSCC patients, a total of 19,467 genes were extracted from the OSCC-TCGA RNA-seq database. It is well-known that stromal and immune cells are two main types of nontumor components in the TME and have been identified to be of value for the diagnosis and prognostic evaluation of tumor. Therefore, the stromal and immune scores of 319 OSCC patients were determined using the ESTIMATE algorithm, and higher immune and stromal scores in the TME resulted in more stromal and immune components in the TME. Furthermore, the ESTIMATE score is the sum of the immune score and the stromal score and represents the combined ratio of the two components in the TME. Thus, these three scores represent the proportion of stromal cells, the proportion of immune cells, and the purity of tumor in the immune microenvironment, respectively [19,26,27]. In the present study, the stromal scores ranged between −1947.438 and 1969.731, immune scores ranged between −392.987 and 2705.005, and ESTIMATE scores ranged between −2340.425 and 4674.735. The OSCC patients were categorized into high and low scores groups based on the stromal, immune, and ESTIMATE scores firstly, and a Kaplan-Meier (KM) survival analysis was used to calculate the OS (overall survival) of OSCC patients. We found that the OS of lower stromal, immune, and ESTIMATE scores of OSCC patients was relatively shorter (Figure 1a–c). Furthermore, based on the clinical data extracted from the TCGA-OSCC database, we found that the stromal, immune, and ESTIMATE scores in T3 and T4 OSCC patients were relatively lower than those in T1 and T2 OSCC patients (Figure 2a–c). Moreover, we also compared the stromal, immune, and ESTIMATE scores with the gender of OSCC patients, and we found that there was no significant difference between stromal, immune, and ESTIMATE scores with genders (Figure 2d–f). We also noticed that there were no significant differences in stromal, immune, and ESTIMATE scores with tumor grades (Figure 2g–i). The above results indicate that stromal, immune, and ESTIMATE scores were relatively associated to the clinical features of OSCC patients. 

### 3.2. Identification of TME-Related Differentially Expressed Genes

**Figure 3:**
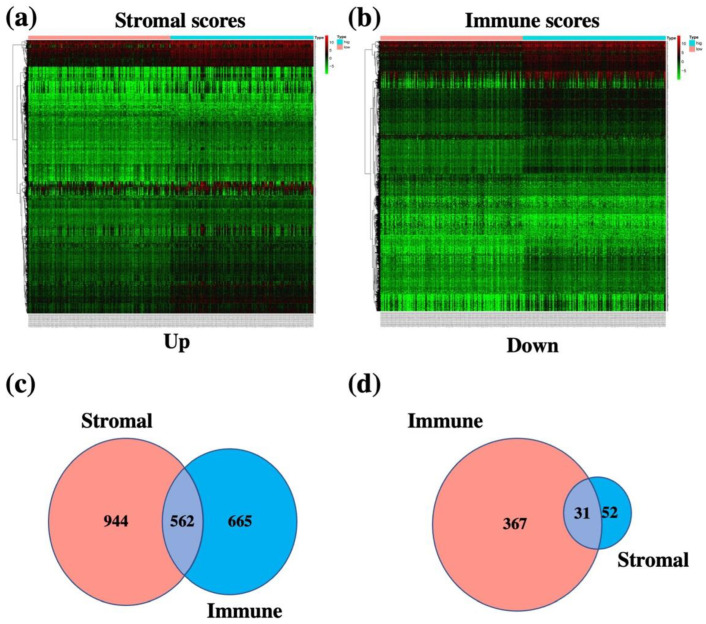
Venn diagram and heatmap analysis of the differentially expressed genes (DEGs) based on the immune and stromal scores. (**a**) Heatmap analysis of the DEGs between the higher stromal scores and lower stromal scores in OSCC patients. (**b**) Heatmap analysis of the DEGs between the higher immune scores and lower immune scores in OSCC patients. (**c**) Venn diagrams analysis the number of upregulated genes of higher stromal scores in OSCC patients and upregulated genes of higher immune scores in OSCC patients. (**d**) Venn diagrams analysis of the number of downregulated genes of higher stromal scores in OSCC patients and downregulated genes of higher immune scores in OSCC patients.

In order to determine the exact changes in gene expression profiles related to immune and stromal components in TME, we investigated the TME-related differentially expressed genes (DEGs) with high and low immune and stromal scores in OSCC patients with the R package “limma”. A total of 1625 DEGs were obtained from the immune score (high and low score samples), with 1227 genes upregulated and 398 genes downregulated (Figure 3a,c,d). Similarly, 1589 DEGs were obtained from the stromal score, with 1506 upregulated genes and 83 downregulated genes (Figure 3b–d). A Venn diagram analysis showed that there were 562 upregulated genes shared by high scores and 31 downregulated genes shared by low scores in immune and stromal scores, and these DEGs (a total of 593 genes) might be the determinants of TME status (Figure 3c,d). These data indicated that TME-related DEGs had been obtained and identified. 

### 3.3. Functional Annotation of TME-Related DEGs

**Figure 4:**
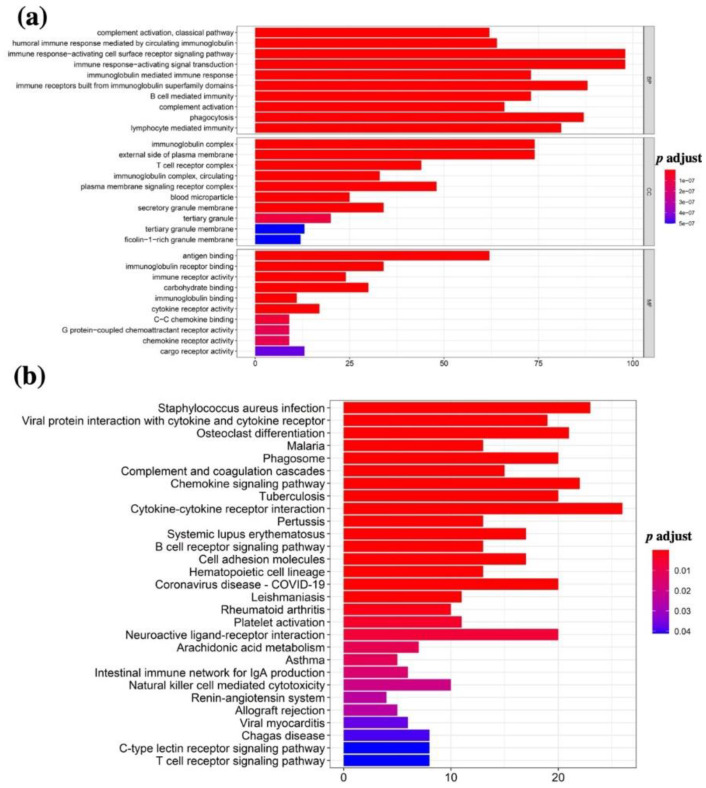
Gene enrichment analysis of the DEGs based on the immune and stromal scores in OSCC patients. (**a**) Gene ontology (GO) enrichment analysis of DEGs based on the immune and stromal scores in OSCC patients. Top panel is the biological process analysis of GO enrichment. Middle panel is the cellular component analysis of GO enrichment. Bottom panel is the molecular function analysis of GO enrichment. (**b**) Kyoto Encyclopedia of Genes and Genomes (KEGG) pathway analysis of DEGs based on the immune and stromal scores in OSCC patients.

To explore the biological functions of the above TME-related DEGs, GO and KEGG enrichment analyses were performed using the R package “cluster Profiler”. As shown in Figure 4a, the top 10 GO terms of the up- and downregulated DEGs were related to different biological processes (BP), cellular components (CC), and molecular functions (MF). Interestingly, the top five terms related to the biological process included lymphocyte activation, regulation of lymphocyte-mediated immunity, phagocytosis, complement activation, and B cell-mediated immunity (Figure 4a, top panel). In addition, the top five terms related to a cellular component included immunoglobulin complex, external side of plasma membrane, plasma membrane signaling receptor complex, T cell receptor, and immunoglobulin complex and circulating (Figure 4a, middle panel). The top five terms related to molecular functions included antigen binding, immunoglobulin receptor binding, carbohydrate-binding, immune receptor activity, and cytokine receptor activity (Figure 4a, bottom panel). On the other hand, the top 30 KEGG pathways for the DEGs related to the up- and downregulated DEGs included immune/inflammation-related pathways, including B cell receptor signaling pathway, T cell receptor signaling pathway, intestinal immune network for IgA production, natural-killer-cell-mediated cytotoxicity, and C-type lectin receptor signaling pathway (Figure 4b). The above data indicate that the signaling pathways and biological behaviors enriched by immune-related DEGs are related to the tumor microenvironment or immune functions.

### 3.4. Protein-Protein Interaction (PPI) Analysis of the TME-Related DEGs

**Figure 5:**
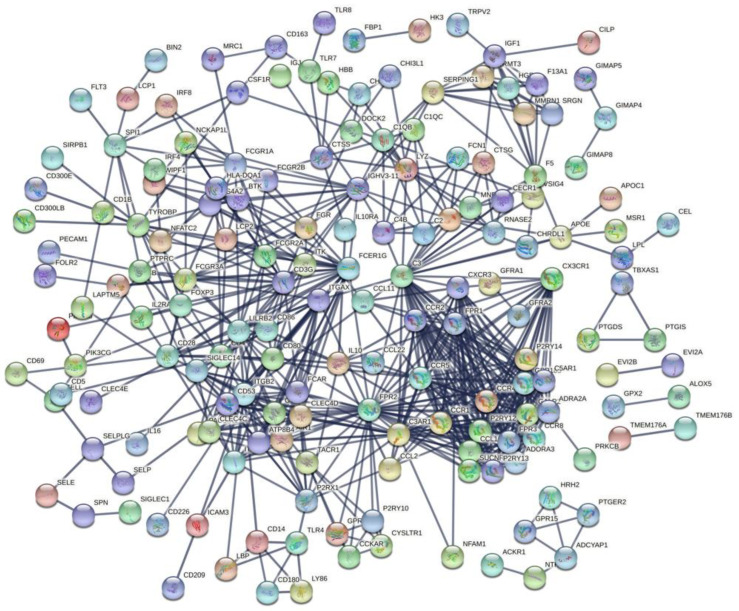
The protein-protein interaction (PPI) analysis of DEGs based on the immune and stromal scores in OSCC patients. The interaction nodes, scores > 0.95 were set as the cutoff to the PPIs network.

**Figure 6:**
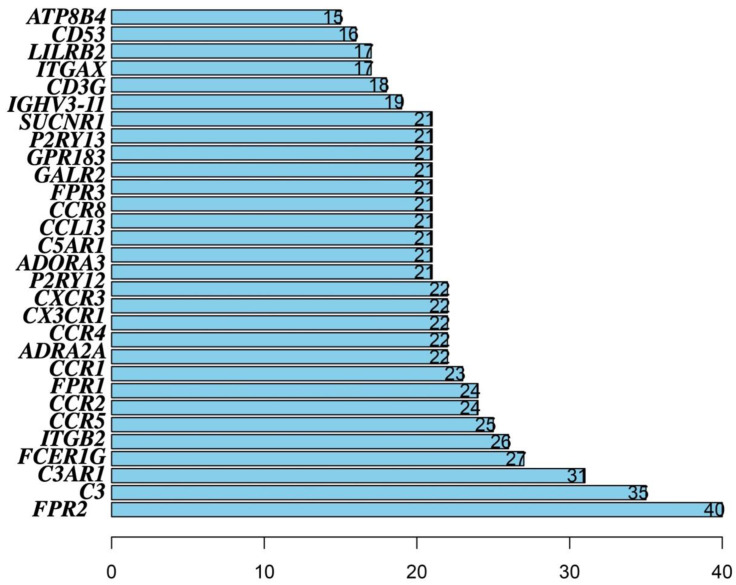
The top 30 key genes analyzed by PPI which associated with the prognosis of OSCC patients, including *ATP8B4*, *CD53*, *LILRB2*, *ITGAX*, *CD3G*, *IGHV3-11*, *SUCNR1*, *P2RY13*, *GPR183*, *GALR2*, *FPR3*, *CCR8*, *CCL13*, *C5AR1*, *ADORA3*, *P2RY12*, *CXCR3*, *CX3CR1*, *CCR4*, *ADRA2A*, *CCR1*, *FPR1*, *CCR2*, *CCR5*, *ITGB2*, *FCER1G*, *C3AR1*, *C3* and *FPR2*.

To study the interaction between DEGs, and systematically conduct a comprehensive study on the crossover gene, all 167 intersection genes were uploaded to the Search Tool Retrieval of Interacting Genes (STRING) database to construct a PPI network. In our study, CYTOSCAPE software was used to construct a PPI network based on the STRING database, and the interaction between 593 DEGs (in Figure 3c,d, there were 562 upregulated genes shared by a high score and 31 downregulated genes shared by a low score in immune and stromal scores, and a total of 593 DEGs) was analyzed. Then, we performed a univariate COX regression analysis to identify that 23 genes (identified by PPI analysis) were strongly associated with OSCC patients’ survival, and each of these 23 genes was independently associated with OSCC patients’ prognosis (Figure 5). The top 30 genes in the network and each node’s number included *ATP8B4*, *CD53*, *LILRB2*, *ITGAX*, *CD3G*, *IGHV3-11*, *SUCNR1*, *P2RY13*, *GPR183*, *GALR2*, *FPR3*, *CCR8*, *CCL13*, *C5AR1*, *ADORA3*, *P2RY12*, *CXCR3*, *CX3CR1*, *CCR4*, *ADRA2A*, *CCR1*, *FPR1*, *CCR2*, *CCR5*, *ITGB2*, *FCER1G*, *C3AR1*, *C3,* and *FPR2* (Figure 6).

### 3.5. Construction of the Risk Score Prognosis Model 

**Figure 7:**
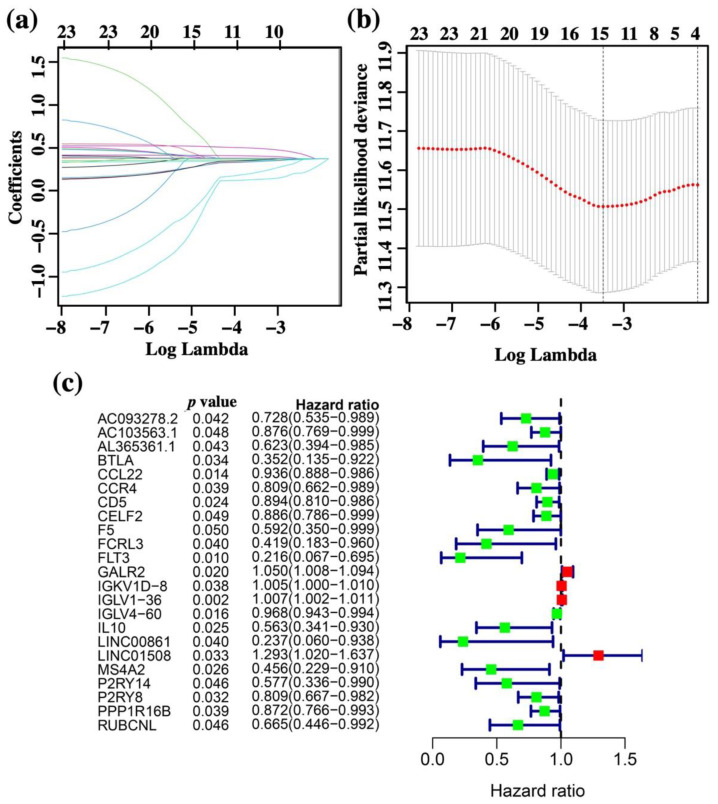
Univariate Cox and LASSO Cox survival analysis for the DEGs based on PPI analysis in OSCC patients. (**a**) Univariate Cox survival analysis of the DEGs. (**b**,**c**) LASSO Cox analysis identified 23 DEGs with the prognostic value and 23 DEGs were identified as prognostic factors of OSCC patients. Protective factors mean the genes’ HR was <1, while risk factors mean the genes’ HR was >1 in OSCC patients.

To analyze the survival-related immune-DGEs, univariate Cox proportional hazards regression model survival analyses were performed, and a total of 23 prognostic genes were identified as survival-related DEGs: *AC093278.2, AC103563.1, AL365361.1, BTLA, CCL22, CCR4, CD5, CELF2, F5, FCRL3, FLT3, GALR2, IGKV1D-8, IGLV1-36, IGLV4-60, IL10, LINC00861, LINC01508, MS4A2, P2RY14, P2RY8, PPP1R16B,* and *RUBCNL* (Figure 7). The least absolute shrinkage and selection operator (LASSO) was then used to exclude the overfitting false positives data (Figure 7a,b). Finally, a prognostic model with 11 survival-related DEGs was constructed for predicting the OS of OSCC patients. The risk scores were calculated for each patient as follows: risk score = −0.0418 × (expression of *AC103563.1*) − 0.0289 × (expression of *CCL22*) − 0.2829 × (expression of *FLT3*) + 0.03681 × (expression of *GALR2*) + 0.0048 × (expression of *IGKV1D.8*) + 0.0067 × (expression of *IGLV1.36*) − 0.0139 × (expression of *IGLV4.60*) − 0.0456 × (expression of *IL10*) − 0.2040 × (expression of *LINC00861*) + 0.1421 × (expression of *LINC01508*) − 0.0245 × (expression of *MS4A2*). The median value was taken as the cutoff, the OSCC patients with higher risk scores than the median value were classified in the high-risk group, and the OSCC patients with lower risk scores than the median value were classified in the low-risk group.

### 3.6. Survival Analysis of the 11-Gene Immune-Related Signature

**Figure 8:**
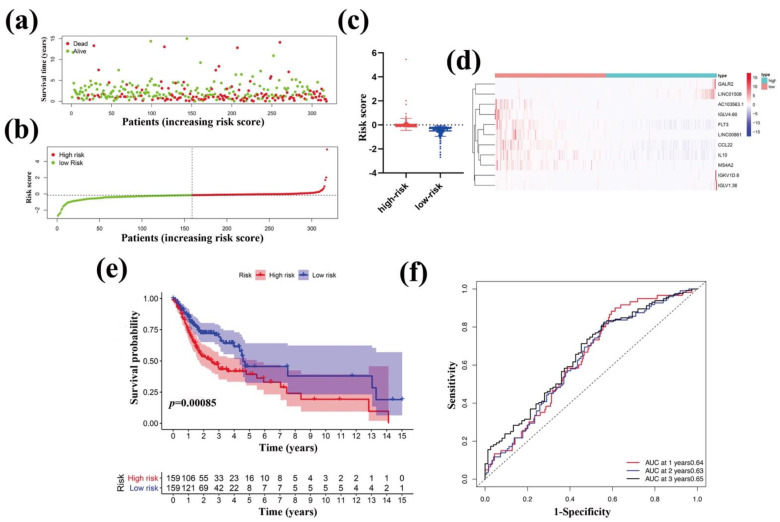
Prognostic effect of the immune-related signature. The survival status (**a**), risk scores (**b**,**c**) of OSCC patients, and heatmap of immune-related DEGs expression pattern (**d**). Kaplan-Meier survival curve (**e**) for OS of the low-risk and high-risk groups of OSCC patients. (**f**) Prognostic value evaluation of the 11-gene signature using time-specific ROC curves and dynamic AUC lines analysis. The time-dependent ROC curves are based on 1, 2, and 3 years of follow-up and the dynamic AUC lines of OSCC patients.

To test the effect of the above model on the evaluation of OSCC patients in the future, we analyzed the outcome survival (OS) of the high-risk group and the low-risk group of OSCC patients. The risk distribution, survival status, and gene expression pattern are shown in Figure 8a–c. The scatter plot (Figure 8a) shows that most of the patients in the high-risk score group died and most of the patients in the low-risk group survived during 15 years of follow-up. Moreover, the gene expression pattern (Figure 8b,c) shows that the high- and low-risk groups we categorized had corresponding risk scores. The heatmap (Figure 8d) showed that seven immune-related DEGs (AC103563.1, CCL22, FLT3, IGLV4.60, IL10, LINC00861, and MS4A2) were highly expressed in the low-risk group while four immune-related DEGs (GALR2, LINC01508, IGKV1D.8 and IGLV1.36) were highly expressed in the high-risk group. To determine the relationship between immune-related DEGs and OS in OSCC patients, we used the Kaplan-Meier method to plot the survival curves using data obtained from the OSCC-TCGA database. The Kaplan-Meier plots showed that patients in the high-risk score group had a significantly poorer OS than those in the low-risk score group (Figure 8e). Moreover, the AUC for 1-year, 2-year, and 3-year PFS were 0.64, 0.63, and 0.65, respectively (Figure 8f). Thus, these data suggested that the 11-gene immune-related signature performed well for predicting OS in OSCC patients.

### 3.7. Validation of an External GEO Cohort

**Figure 9:**
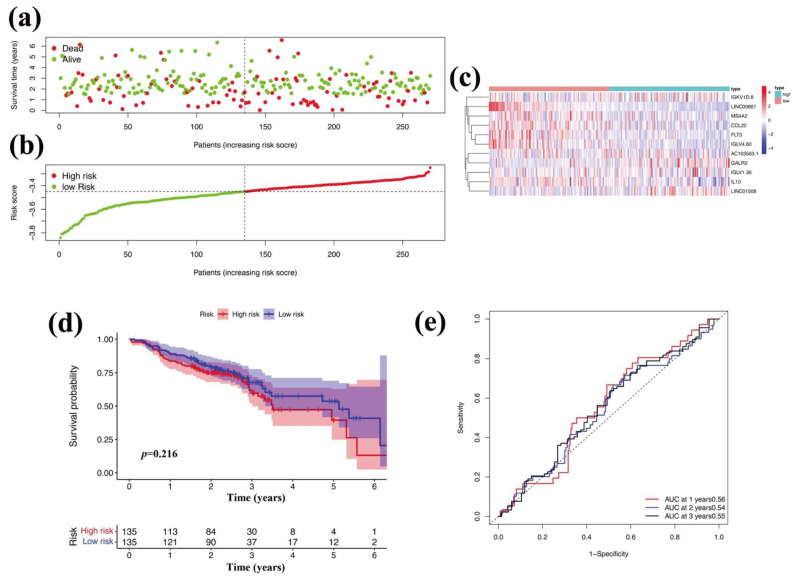
Validation of the 11-gene signature in the GEO cohort (GSE65858). The overall survival status (**a**), risk scores (**b**), the heatmap of the 11-gene expression pattern (**c**), Kaplan-Meier curve analysis with risk scores (**d**), and ROC curve (**e**) are shown.

To verify the clinical value of the 11-gene TME-related signature for predicting OS in OSCC patients, we used an external GEO-OSCC cohort (GSE65858) to validate our study. The 11-gene TME-related signature was constructed in GSE65858, and the risk scores were analyzed by the same methods. High- and low-risk OSCC groups were classified according to the median risk score. A Kaplan-Meier curve analysis showed that the low-risk score was closely associated with a longer overall survival time (Figure 9d), which was consistent with that the TCGA cohort (Figure 8d). In addition, the ROC curve showed an AUC value was 0.55 at 1, 2, and 3 years (Figure 9e), which was consistent with that the TCGA cohort (Figure 8e). Thus, the results showed that the 11-gene TME-related signature could effectively predict the prognosis of OSCC patients partly.

## 4. Discussion

In the present study, differentially expressed immune-related genes in oral squamous carcinoma cancer (OSCC) samples were identified and were used to construct a signature with 11 immune-related genes, which performed well in predicting the outcomes of OSCC patients, respectively.

OSCC ranks as the eighth most common form of oral malignancy, with an estimated 500,000 new cases being reported annually, and OSCC is associated with a high grade, rapid progression, poor treatment effects, and bad outcomes [28]. The clinical treatment of OSCC is often a combination of surgical resection with chemotherapy; however, the effect of these treatments is very limited and the 5-year survival rate of OSCC patients remains lower than 50% [29,30]. There are several ways to predict the prognosis of OSCC patients, including but not limited to the following: (1) TNM stages; (2) tumor grade; (3) the number of lymph node metastasis; (4) the expression level of key proteins, such as epidermal growth factor receptor and P53 [31,32,33]. However, a unified and effective standard still lacks to determine the prognosis of OSCC patients. 

The TME is the cellular environment in which tumor cells live, which consists of an extracellular matrix, soluble molecules, and tumor stromal cells [10]. The TME plays a vital role in OSCC progression and consists of tumor cells and other components, such as immune cells, stromal scores, tumor-associated macrophages (TAMs), and carcinoma-associated fibroblasts (CAFs) [34,35]. Moreover, tumor development, progression, and responses to immunotherapies are regulated by cytokines, immune infiltration, TAMs, and CAFs within the TME [36,37]. It is well known that immune cells and stromal cells are two main types of nontumor components in the TME and have been proposed to be of value for the diagnosis and prognostic evaluation of tumors [38]. Recently, ESTIMATE has been used as a tool for inferring immune, stromal, and immune scores in the TME, and the estimation is based on the expression signals of gene sets that characterize stromal and immune cells. Therefore, in this study, we developed an immune-related signature based on stromal and immune scores to predict the prognosis of OSCC patients, which could improve the response rate to immunotherapy. In addition, ESTIMATE is an algorithm that has been used to calculate the immune and stromal scores in several cancers, including pancreatic ductal adenocarcinoma [39], colorectal cancer [40], and OSCC [17,21,22].

In the present study, we conducted a comprehensive analysis between the immune cells and immune-related genes and the clinical indexes and outcomes of OSCC patients. We found the following findings: (1) The clinical indexes (such as TNM stages, grade stages, and genders) of OSCC patients were associated with the stromal, immune, and ESTIMATE scores, but the correlation was not significant (Figure 1). However, high stromal, immune, and ESTIMATE scores correlated with a relatively longer OS of OSCC patients, indicating that the TME components, especially the stromal and immune cells affected the clinical outcomes of OSCC patients (Figure 2). (2) In total, 593 DEGs were identified from the comparison of high versus low stromal and immune scores groups based on the median value of immune and stromal scores as the standard cutoff to divide patients into high-score and low-score groups. Then, the results of GO terms showed that the DEGs in the high-score group regulated the T cell receptor complex and immunoglobulin complex (Figure 3a). Moreover, GO and KEGG pathway enrichment data indicated that the DEGs in the high-score group also regulated the T cell receptor signaling pathway and viral myocarditis (Figure 3b). (3) LASSO and random forest (RF) algorithm analyses identified the 11 prognostic immune-related genes (AC103563.1, CCL22, FLT3, GALR2, IGKV1D.8, IGLV1.36, IGLV4.60, IL10, LINC00861, LINC01508, and MS4A2) (summarized in Tables S1 and S2). In particular, high levels of GALR2, LINC01508, IGKV1D.8, and IGLV1.36 were found to be negatively correlated with the OS of OSCC patients, while high levels of AC103563.1, CCL22, FLT3, IGLV4.60, IL10, LINC00861, and MS4A2 were positively correlated with the OS of OSCC patients. (4) To verify the clinical value of the 11-gene immune-related signature for predicting OS in OSCC patients, we used an external GEO-OSCC cohort (GEO65858) for the validation in our study. Altogether, the results showed that the 11-gene TME-related signature could effectively predict the prognosis of OSCC patients.

The clinical implications of these 11 key genes have been reported in different types of cancers. For instance, CCL22 (C-C motif chemokine 22) controls T cell immunity by recruiting regulatory T cells to the tumor tissue and promoting regulatory T cell communication with dendritic cells in TME [41]. Higher infiltration rates of CCL22+ cells are reported to be associated with poor outcomes in cervical cancer patients [42]. Moreover, studies have shown the efficacy of flt3-L and CD40-L combination immunotherapy on prostate tumor growth in TME [43]. Moreover, FLT3 is a potential targeted protein and has been reported as an immune-related prognostic gene in lung adenocarcinoma [44], indicating the clinical value of FLT3. Furthermore, galanin receptor 2 (GALR2) is a G protein-coupled receptor that induces tumor growth and proliferation in SCCHN (squamous cell carcinoma of the head and neck) [45], and GALR2 has been identified as a novel target and biomarker for prostate, colon, and breast cancer screening [46]. In addition, IL10 is an important immune regulatory cytokine in TME [47], and a recent study has shown that IL10 promotes cancer cell metastasis and proliferation via immunosuppression, and also has a predictive value in clear cell renal cell carcinoma [48]. Recently, a study has shown that the MS4A2 (membrane-spanning 4 domains, superfamily A, number 2) gene is located on chromosome 11q13, a region that is linked to asthma-related phenotypes, and it plays an important role in the regulation of human mast cell proliferation and survival [49]. However, very few studies report the role of MAS4A2 in TME. Other immune-related genes are relatively novel and scattered evidence has been reported for their roles in human diseases. For example, IGLV1-36 gene is a potentially therapeutic option for POEMS (polyneuropathy, organomegaly, endocrinopathy, monoclonal gammopathy, and skin changes) syndrome [50]. Moreover, the downregulation of LINC01508, a long noncoding RNAs, contributes to cisplatin resistance in ovarian cancer [51]. Additionally, another recent study has shown that the LINC00861/miR-513b-5p axis inhibits cancer cell progression through the PTEN/AKT/mTOR signaling pathway in cervical cancer cells [52]. Furthermore, LINC00861 is also used as a biomarker to predict survival in patients with ovarian cancer [53], the early diagnosis of Parkinson’s disease [54], and breast cancer [55]. However, few studies have reported the role of AC103563.1, IGKV1D.8, and IGLV4.60 in TME (especially in OSCC), which will be our future directions.

Similar studies have also been shown in OSCC. For instance, Li et al. selected eleven immunosuppression genes (ISGs), including INHBB, BGLAP, CTLA4, CALCA, CXCL8, IL22, FGFR3, HPRT1, ORMDL3, SPHK1, and TLR3, which showed a prognostic potential for OSCC [21]. In this study, a deep-learning-based model showed two subgroups of OSCC samples, while subtype Sub1 displayed a more aggressive phenotype with poor prognosis, with immune-cells-associated pathways and enriched cancer-progression-pertinent pathways. In addition, to test if the stemness and immune-relevant genes were involved in oral cancer, Lin et al. established an eight-gene risk model (ESCO2, CCNA2, COL5A3, RCN3, LMCD1, FMNL3, MMP14, and HEYL), which performed well in predicting overall survival and recurrence-free survival in OSCC patients [17]. Further investigations showed that the eight-gene signature was highly linked to immune suppression. However, we used different methods to independently establish our gene signature, including a protein—protein interaction analysis, GO and a KEGG enrichment analysis. More importantly, our 11-gene signature involved either LincRNA and a few novel genes, which could potentially contribute to oral cancer progression and treatment resistance in the TME. Altogether, our studies and others have all provided potentially promising ways to obtain a prognosis for OSCC patients.

However, our study still has limitations. For example, the OSCC TME-related signature was not verified by biological experiments both in vivo and in vitro, but in our ongoing projects, these immune-related genes and their potential mechanisms in OSCC are being verified. Moreover, due to the limited samples in the OSCC subgroups, the prognostic value of the immune-related signature in some OSCC subgroups did not show any statistical significance. Therefore, a large cohort of OSCC patient samples is needed for future validations. 

## 5. Conclusions

In summary, we proposed a signature of 11 immune-related genes based on the TME in OSCC, which could be used as an independent prognostic biomarker for OSCC patients. The signature of 11 immune-related genes had a predominant performance in obtaining the prognosis of OSCC patients, and it could achieve a more personalized and precise immunotherapy effect in OSCC.

## Data Availability

Not applicable.

## Supplementary Materials

The following supporting information can be downloaded at: https://www.mdpi.com/article/10.3390/vaccines10091521/s1, Table S1: immune-all, Table S2: stromal-all, Text S1: R code.

## References

[1] Siegel R.L., Miller K.D., Jemal A.V. (2020). Cancer statistics, 2020. CA Cancer J. Cliv..

[2] Yang Z., Yan G., Zheng L., Gu W., Liu F., Chen W., Cui X., Wang Y., Yang Y., Chen X. (2021). YKT6, as a potential predictor of prognosis and immunotherapy response for oral squamous cell carcinoma, is related to cell invasion, metastasis, and CD8+ T cell infiltration. Oncoimmunology.

[3] Ho A.S., Kim S., Tighiouart M., Gudino C., Mita A., Scher K.S., Laury A., Prasad R., Shiao S.L., Van Eyk J.E. (2017). Metastatic Lymph Node Burden and Survival in Oral Cavity Cancer. J. Clin. Oncol..

[4] Sasahira T., Kirita T. (2018). Hallmarks of Cancer-Related Newly Prognostic Factors of Oral Squamous Cell Carcinoma. Int. J. Mol. Sci..

[5] Almangush A., Makitie A.A., Triantafyllou A., de Bree R., Strojan P., Rinaldo A., Hernandez-Prera J.C., Suarez C., Kowalski L.P., Ferlito A. (2020). Staging and grading of oral squamous cell carcinoma: An update. Oral Oncol..

[6] Sa J.O., Trino L.D., Oliveira A.K., Lopes A.F.B., Granato D.C., Normando A.G.C., Santos E.S., Neves L.X., Carnielli C.M., Paes Leme A.F. (2021). Proteomic approaches to assist in diagnosis and prognosis of oral cancer. Expert Rev. Proteom..

[7] Chai A.W.Y., Lim K.P., Cheong S.C. (2020). Translational genomics and recent advances in oral squamous cell carcinoma. Semin. Cancer Biol..

[8] Alves A.M., Diel L.F., Lamers M.L. (2018). Macrophages and prognosis of oral squamous cell carcinoma: A systematic review. J. Oral Pathol. Med..

[9] Li Q., Xia D., Wang Z., Liu B., Zhang J., Peng P., Tang Q., Dong J., Guo J., Kuang D. (2021). Circadian Rhythm Gene PER3 Negatively Regulates Stemness of Prostate Cancer Stem Cells via WNT/beta-Catenin Signaling in Tumor Microenvironment. Front. Cell Dev. Biol..

[10] Liu J., Lu J., Li W. (2022). Transcriptome analysis reveals the prognostic and immune infiltration characteristics of glycolysis and hypoxia in head and neck squamous cell carcinoma. BMC Cancer.

[11] Albini A., Sporn M.B. (2007). The tumour microenvironment as a target for chemoprevention. Nat. Rev. Cancer.

[12] Castells M., Thibault B., Delord J.P., Couderc B. (2012). Implication of tumor microenvironment in chemoresistance: Tumor-associated stromal cells protect tumor cells from cell death. Int. J. Mol. Sci..

[13] Wang G., Liu P., Li J., Jin K., Zheng X., Xie L. (2022). Novel Prognosis and Therapeutic Response Model of Immune-Related lncRNA Pairs in Clear Cell Renal Cell Carcinoma. Vaccines.

[14] Dong P., Xiong Y., Yue J., Hanley S.J.B., Watari H. (2018). B7H3 As a Promoter of Metastasis and Promising Therapeutic Target. Front. Oncol..

[15] Joshi P.A., Waterhouse P.D., Kasaian K., Fang H., Gulyaeva O., Sul H.S., Boutros P.C., Khokha R. (2019). PDGFRalpha(+) stromal adipocyte progenitors transition into epithelial cells during lobulo-alveologenesis in the murine mammary gland. Nat. Commun..

[16] Taber A., Christensen E., Lamy P., Nordentoft I., Prip F., Lindskrog S.V., Birkenkamp-Demtroder K., Okholm T.L.H., Knudsen M., Pedersen J.S. (2020). Molecular correlates of cisplatin-based chemotherapy response in muscle invasive bladder cancer by integrated multi-omics analysis. Nat. Commun..

[17] Lin X., Zheng X., Yang B., Chen J., Xu Q., Wang Q. (2021). Clinical significance and immune landscapes of stemness-related and immune gene set-based signature in oral cancer. Clin. Transl. Med..

[18] Smyth M.J., Ngiow S.F., Ribas A., Teng M.W. (2016). Combination cancer immunotherapies tailored to the tumour microenvironment. Nat. Rev. Clin. Oncol..

[19] Yoshihara K., Shahmoradgoli M., Martinez E., Vegesna R., Kim H., Torres-Garcia W., Trevino V., Shen H., Laird P.W., Levine D.A. (2013). Inferring tumour purity and stromal and immune cell admixture from expression data. Nat. Commun..

[20] Job S., Rapoud D., Dos Santos A., Gonzalez P., Desterke C., Pascal G., Elarouci N., Ayadi M., Adam R., Azoulay D. (2020). Identification of Four Immune Subtypes Characterized by Distinct Composition and Functions of Tumor Microenvironment in Intrahepatic Cholangiocarcinoma. Hepatology.

[21] Li S., Mai Z., Gu W., Ogbuehi A.C., Acharya A., Pelekos G., Ning W., Liu X., Deng Y., Li H. (2021). Molecular Subtypes of Oral Squamous Cell Carcinoma Based on Immunosuppression Genes Using a Deep Learning Approach. Front. Cell Dev. Biol..

[22] Zhang S.Y., Ren X.Y., Wang C.Y., Chen X.J., Cao R.Y., Liu Q., Pan X., Zhou J.Y., Zhang W.L., Tang X.R. (2021). Comprehensive Characterization of Immune Landscape Based on Epithelial-Mesenchymal Transition Signature in OSCC: Implication for Prognosis and Immunotherapy. Front. Oncol..

[23] Ritchie M.E., Phipson B., Wu D., Hu Y., Law C.W., Shi W., Smyth G.K. (2015). Limma powers differential expression analyses for RNA-sequencing and microarray studies. Nucl. Acids Res..

[24] Yu G., Wang L.G., Han Y., He Q.Y. (2012). Clusterprofiler: An R package for comparing biological themes among gene clusters. OMICS.

[25] Szklarczyk D., Gable A.L., Lyon D., Junge A., Wyder S., Huerta-Cepas J., Simonovic M., Doncheva N.T., Morris J.H., Bork P. (2019). STRING v11: Protein-protein association networks with increased coverage, supporting functional discovery in genome-wide experimental datasets. Nucl. Acids Res..

[26] Wang Z., Wang Y., Yang T., Xing H., Wang Y., Gao L., Guo X., Xing B., Wang Y., Ma W. (2021). Machine learning revealed stemness features and a novel stemness-based classification with appealing implications in discriminating the prognosis, immunotherapy and temozolomide responses of 906 glioblastoma patients. Brief. Bioinform..

[27] Zhao B., Wang Y., Wang Y., Chen W., Liu P.H., Kong Z., Dai C., Wang Y., Ma W. (2021). Systematic identification, development, and validation of prognostic biomarkers involving the tumor-immune microenvironment for glioblastoma. J. Cell Physiol..

[28] Elbers J.B.W., Al-Mamgani A., Paping D., van den Brekel M.W.M., Jozwiak K., de Boer J.P., Karakullukcu B., Verheij M., Zuur C.L. (2017). Definitive (chemo)radiotherapy is a curative alternative for standard of care in advanced stage squamous cell carcinoma of the oral cavity. Oral Oncol..

[29] Warnakulasuriya S. (2009). Global epidemiology of oral and oropharyngeal cancer. Oral Oncol..

[30] Chen T.W., Lee C.C., Liu H., Wu C.S., Pickering C.R., Huang P.J., Wang J., Chang I.Y., Yeh Y.M., Chen C.D. (2017). APOBEC3A is an oral cancer prognostic biomarker in Taiwanese carriers of an APOBEC deletion polymorphism. Nat. Commun..

[31] Amit M., Takahashi H., Dragomir M.P., Lindemann A., Gleber-Netto F.O., Pickering C.R., Anfossi S., Osman A.A., Cai Y., Wang R. (2020). Loss of p53 drives neuron reprogramming in head and neck cancer. Nature.

[32] van Roessel S., Kasumova G.G., Verheij J., Najarian R.M., Maggino L., de Pastena M., Malleo G., Marchegiani G., Salvia R., Bassi S.C. (2018). International Validation of the Eighth Edition of the American Joint Committee on Cancer (AJCC) TNM Staging System in Patients With Resected Pancreatic Cancer. JAMA Surg..

[33] Li Q., Wang J., Meng X., Chen W., Feng J., Mao J. (2021). Identification of autophagy-related gene and lncRNA signatures in the prognosis of HNSCC. Oral Dis..

[34] Chen J.H., Wu A.T.H., Bamodu O.A., Yadav V.K., Chao T.Y., Tzeng Y.M., Mukhopadhyay D., Hsiao M., Lee J.C. (2019). Ovatodiolide Suppresses Oral Cancer Malignancy by Down-Regulating Exosomal Mir-21/STAT3/beta-Catenin Cargo and Preventing Oncogenic Transformation of Normal Gingival Fibroblasts. Cancers.

[35] Li Z., Liu F.Y., Kirkwood K.L. (2020). The p38/MKP-1 signaling axis in oral cancer: Impact of tumor-associated macrophages. Oral Oncol..

[36] Dourado M.R., Korvala J., Astrom P., De Oliveira C.E., Cervigne N.K., Mofatto L.S., Campanella Bastos D., Pereira Messetti A.C., Graner E., Paes Leme A.F. (2019). Extracellular vesicles derived from cancer-associated fibroblasts induce the migration and invasion of oral squamous cell carcinoma. J. Extracell. Vesicles.

[37] Quan H., Shan Z., Liu Z., Liu S., Yang L., Fang X., Li K., Wang B., Deng Z., Hu Y. (2020). The repertoire of tumor-infiltrating lymphocytes within the microenvironment of oral squamous cell carcinoma reveals immune dysfunction. Cancer Immunol. Immunother..

[38] Hwang I., Kim J.W., Ylaya K., Chung E.J., Kitano H., Perry C., Hanaoka J., Fukuoka J., Chung J.Y., Hewitt S.M. (2020). Tumor-associated macrophage, angiogenesis and lymphangiogenesis markers predict prognosis of non-small cell lung cancer patients. J. Transl. Med..

[39] Kandimalla R., Tomihara H., Banwait J.K., Yamamura K., Singh G., Baba H., Goel A. (2020). A 15-Gene Immune, Stromal, and Proliferation Gene Signature that Significantly Associates with Poor Survival in Patients with Pancreatic Ductal Adenocarcinoma. Clin. Cancer Res..

[40] Fakih M., Ouyang C., Wang C., Tu T.Y., Gozo M.C., Cho M., Sy M., Longmate J.A., Lee P.P. (2019). Immune overdrive signature in colorectal tumor subset predicts poor clinical outcome. J. Clin. Investig..

[41] Flores C.T., Wildes T.J., Drake J.A., Moore G.L., Dean B.D., Abraham R.S., Mitchell D.A. (2018). Lin(-)CCR2(+) hematopoietic stem and progenitor cells overcome resistance to PD-1 blockade. Nat. Commun..

[42] Wang Q., Schmoeckel E., Kost B.P., Kuhn C., Vattai A., Vilsmaier T., Mahner S., Mayr D., Jeschke U., Heidegger H.H. (2019). Higher CCL22+ Cell Infiltration is Associated with Poor Prognosis in Cervical Cancer Patients. Cancers.

[43] Ciavarra R.P., Brown R.R., Holterman D.A., Garrett M., Glass W.F., Wright G.L., Schellhammer P.F., Somers K.D. (2003). Impact of the tumor microenvironment on host infiltrating cells and the efficacy of flt3-ligand combination immunotherapy evaluated in a treatment model of mouse prostate cancer. Cancer Immunol. Immunother..

[44] Daver N., Schlenk R.F., Russell N.H., Levis M.J. (2019). Targeting FLT3 mutations in AML: Review of current knowledge and evidence. Leukemia.

[45] Banerjee R., Van Tubergen E.A., Scanlon C.S., Vander Broek R., Lints J.P., Liu M., Russo N., Inglehart R.C., Wang Y., Polverini P.J. (2014). The G protein-coupled receptor GALR2 promotes angiogenesis in head and neck cancer. Mol. Cancer Ther..

[46] Chung W., Kwabi-Addo B., Ittmann M., Jelinek J., Shen L., Yu Y., Issa J.P. (2008). Identification of novel tumor markers in prostate, colon and breast cancer by unbiased methylation profiling. PLoS ONE.

[47] Vera-Lozada G., Minnicelli C., Segges P., Stefanoff G., Kristcevic F., Ezpeleta J., Tapia E., Niedobitek G., Barros M.H.M., Hassan R. (2018). Interleukin 10 (IL10) proximal promoter polymorphisms beyond clinical response in classical Hodgkin lymphoma: Exploring the basis for the genetic control of the tumor microenvironment. Oncoimmunology.

[48] Wan B., Liu B., Huang Y., Lv C. (2020). Identification of genes of prognostic value in the ccRCC microenvironment from TCGA database. Mol. Genet. Genom. Med..

[49] Cruse G., Kaur D., Leyland M., Bradding P. (2010). A novel FcepsilonRIbeta-chain truncation regulates human mast cell proliferation and survival. FASEB J..

[50] Bender S., Javaugue V., Saintamand A., Ayala M.V., Alizadeh M., Filloux M., Pascal V., Gachard N., Lavergne D., Auroy F. (2020). Immunoglobulin variable domain high-throughput sequencing reveals specific novel mutational patterns in POEMS syndrome. Blood.

[51] Xiao L., Shi X.Y., Li Z.L., Li M., Zhang M.M., Yan S.J., Wei Z.L. (2021). Downregulation of LINC01508 contributes to cisplatin resistance in ovarian cancer via the regulation of the Hippo-YAP pathway. J. Gynecol. Oncol..

[52] Liu H., Zhang L., Ding X., Sui X. (2021). LINC00861 inhibits the progression of cervical cancer cells by functioning as a ceRNA for miR513b5p and regulating the PTEN/AKT/mTOR signaling pathway. Mol. Med. Rep..

[53] Zheng M., Hu Y., Gou R., Nie X., Li X., Liu J., Lin B. (2020). Identification three LncRNA prognostic signature of ovarian cancer based on genome-wide copy number variation. Biomed. Pharmacother..

[54] Lei K., Zhang L., He Y., Sun H., Tong W., Xu Y., Jin L. (2020). Immune-associated biomarkers for early diagnosis of Parkinson’s disease based on hematological lncRNA-mRNA co-expression. Biosci. Rep..

[55] Zhang Y., Li Y., Wang Q., Zhang X., Wang D., Tang H.C., Meng X., Ding X. (2017). Identification of an lncRNAmiRNAmRNA interaction mechanism in breast cancer based on bioinformatic analysis. Mol. Med. Rep..

